# Make the right measurement: Discovery of an allosteric inhibition site for p300-HAT

**DOI:** 10.1063/1.5119336

**Published:** 2019-10-11

**Authors:** Anna S. Gardberg, Annissa J. Huhn, Richard Cummings, Archana Bommi-Reddy, Florence Poy, Jeremy Setser, Valerie Vivat, Francois Brucelle, Jonathan Wilson

**Affiliations:** 1 Drug Discovery, Constellation Pharmaceuticals, Cambridge, Massachusetts 02142, USA; 2 Foghorn Therapeutics, Cambridge, Massachusetts 02142, USA

## Abstract

Histone acetyltransferases (HATs) and histone deacetylases (HDACs) catalyze the dynamic and reversible acetylation of proteins, an epigenetic regulatory mechanism associated with multiple cancers. Indeed, HDAC inhibitors are already approved in the clinic. The HAT paralogs p300 and CREB-binding protein (CBP) have been implicated in human pathological conditions including several hematological malignancies and androgen receptor-positive prostate cancer. Others have reported CoA-competitive inhibitors of p300 and CBP with cell-based activity. Here, we describe 2 compounds, CPI-076 and CPI-090, discovered through p300-HAT high throughput screening screening, which inhibit p300-HAT via binding at an allosteric site. We present the high resolution (1.7 and 2.3 Å) co-crystal structures of these molecules bound to a previously undescribed allosteric site of p300-HAT. Derivatization yielded actionable structure-activity relationships, but the full-length enzymatic assay demonstrated that this allosteric HAT inhibitor series was artifactual, inhibiting only the HAT domain of p300 with no effect on the full-length enzyme.

## I. INTRODUCTION

Epigenetics refers to a broad regulatory system that controls gene expression by modifying chromatin. Chromatin is DNA wrapped around an assembly of proteins called histones. Whether a specific set of genes is turned on or off depends on the action of epigenetic regulators. Epigenetic regulators change the architecture of chromatin, allowing it to adopt an open configuration to facilitate gene expression or, conversely, a closed configuration to suppress gene expression. An open chromatin configuration turns on a gene by allowing access and “readout” of the genetic information stored in DNA. A closed chromatin configuration turns off a gene by preventing access to DNA and results in silencing of gene expression. Various chromatin regulatory mechanisms (multiple classes of reader, writer, and eraser proteins) impact the chromatin structure to alter the transcriptional machinery's access to DNA (Jenuwein and Allis, 2001).

Most epigenetic drug targets fall into three main classes: First, epigenetic writers, which are enzymes that add chemical modifications onto chromatin (e.g., histone acetyltransferases such as p300 and histone methyltransferases such as EZH2); second, epigenetic readers, which are protein families that recognize chemical modifications on chromatin and bind to these modifications using specialized protein domains (e.g., bromodomains); third, epigenetic erasers, which are enzymes that remove chemical modifications from chromatin (e.g., lysine demethylases such as LSD1) (Biswas and Rao, 2018). The action of each class of epigenetic regulators alters the structure of chromatin and ultimately controls transcription.

**FIG. 1.**
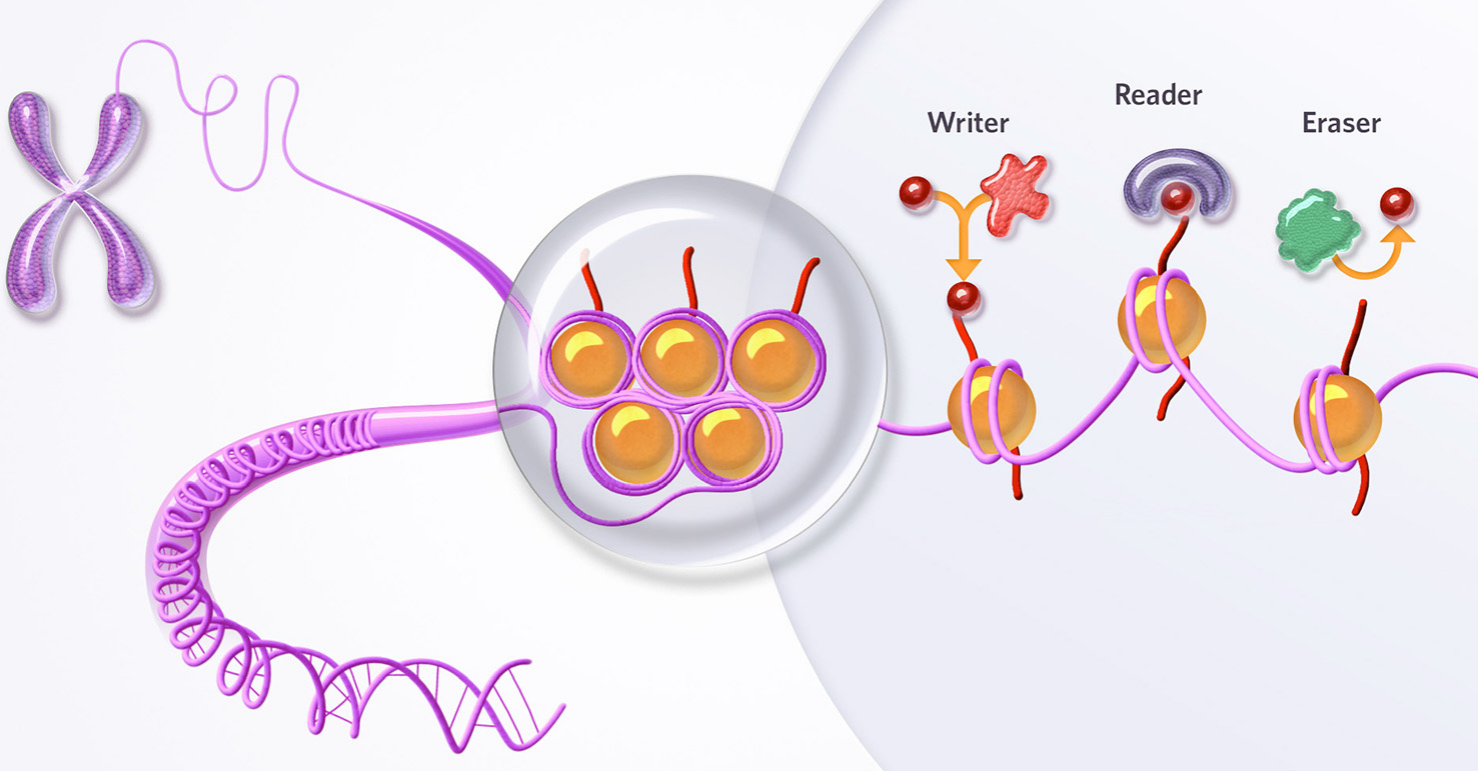
Three classes of epigenetic regulators. Writers add chemical modifications onto chromatin. Readers recognize chemical modifications on chromatin and bind to them. Erasers remove chemical modifications from chromatin. The action of each class controls the expression of certain genes in different ways. Reproduced with permission from Constellation's website.

As illustrated in Fig. 1, epigenetic regulators within the writer, reader, and eraser classes modify chromatin and affect gene expression by adding, binding to, or removing chemical tags, which are indicated by dots, on chromatin. In normal cells, these epigenetic mechanisms are tightly regulated so that genes are “turned on” or “turned off” as appropriate.

Abnormal cells, such as proliferating cancer cells, can dysregulate these epigenetic mechanisms, ultimately leading to disease (Yoo and Jones, 2006). Epigenetic inhibition is particularly attractive as a therapeutic approach for several reasons. (1) Small-molecule inhibitors can block the abnormal function of epigenetic regulators that cancer cells depend on for growth and potentially restore normal gene expression. (2) Cells use hundreds of epigenetic regulators to control gene expression, which provides a large number of potential drug targets.

One epigenetic writer/eraser pair is HAT/KAT and HDAC. Histone or lysine acetyltransferases (HATs/KATs) and histone deacetylases (HDACs) catalyze the dynamic and reversible acetylation of histone proteins, an epigenetic regulatory mechanism associated with multiple cancers. While several HDAC inhibitors are already approved, relatively little is known about HAT inhibitors (Struhl, 1998).

The HAT paralogs p300 and CREB-binding protein (CBP) have been implicated in human pathological conditions (Dutta *et al.,* 2016) including several hematological malignancies and androgen receptor-positive prostate cancer. The focus of this work is the inhibition of histone acetyltransferase p300, a master transcriptional regulator. P300 is an ∼2400AA multidomain protein whose catalytic core consists of bromo-, RING, PHD, and histone acetyltransferase (HAT) domains. By acetylating histones, p300 promotes gene expression. It can also acetylate itself, as well as other proteins, and recruit a larger complex of proteins including transcription factors. The opportunity to target these multiple functions offers broad therapeutic potential in oncology (Lasko *et al.,* 2017).

## II. HTS

To identify starting points for modulating p300/CBP activity, high throughput screening was carried out using the WT P300 HAT domain (WT HAT construct = K1287-F1666 including the L1520-L1580 regulatory loop, expressed as HisTev fusion) and a peptide substrate in a catalytic assay. After extensive triage, several validated inhibitor types were identified, one of which will be the focus of this report. A more detailed description of the HTS efforts will be the subject of a forthcoming publication by Huhn *et al.* At this stage of the project, only the HAT domain of p300 was used, as it was not yet feasible to obtain sufficient quantities of full-length P300 (FL-P300) to conduct a high-throughput screen of >150,000 compounds.

Two different chemotypes, CPI-076 and CPI-090 (Table I), showed a common mechanism of inhibition, with both displaying weaker inhibition at high concentrations of either a peptide substrate or AcCoA. This mechanism initially appeared to be mixed inhibition because a shift in IC50 was seen for both excess substrate MOI tests. On further examination, neither the 10× AcCoA nor the 30× histone peptide met the IC50 shift threshold necessary to be considered truly competitive. In both situations, CPI-076 and CPI-090 are AcCoA- and peptide-modulating compounds but not wholly competitive compounds.

**TABLE I.** Allosteric P300 HAT inhibitor HTS hits.

	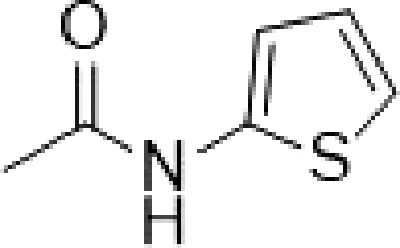	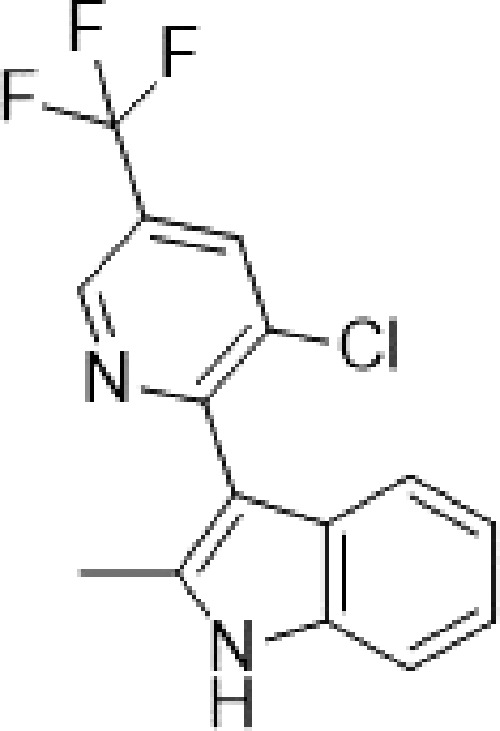
*CPI-number*	CPI-076	CPI-090
*Primary assays*		
p300 HAT SPA IC_50_CBP HAT SPA IC_50_	20 *μ*M 39 *μ*M	0.31 *μ*M 0.74 *μ*M
DSF ΔTm @ 250 *μ*M	+1.3 °C	+5.8 °C

Additionally, given their small size, it seemed unlikely that CPI-076 and CPI-090 blocked both sites. Followup with dynamic scanning fluorimetry (DSF) of the P300-HAT domain indicated strong stabilization, especially for CPI-090. As detailed below, crystallography was key to unraveling these observations by revealing that these inhibitors occupied a previously undescribed allosteric pocket.

## III. STRUCTURE DETERMINATION AND SBDD

### Crystallization methods

To improve homogeneity, a construct omitting the autoacetylation-prone autoinhibitory loop was inactivated through mutation of residue Y1467 to F: p300‐HAT‐YF‐Δloop [K1287‐Q1663/Y1467F/Δ(L1520‐L1580)] and overexpressed in *E. coli*. The enzyme was purified by Nickel Sepharose and ion-exchange chromatography. Initial crystals were obtained with CoA by vapor diffusion crystallization. From these, stocks of microseeds were prepared.

**FIG. 2.**
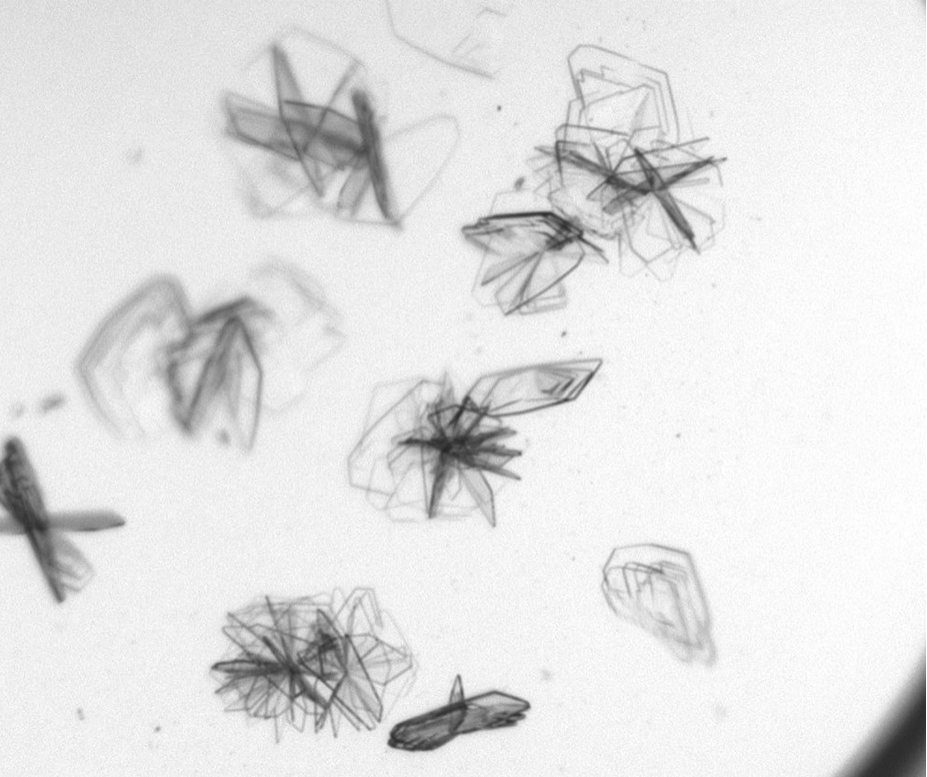
Crystals of p300‐HAT‐YF‐Δloop with CoA and CPI-090.

Blade-form ternary crystals of p300‐HAT‐YF‐Δloop with CoA and CPI-076 were obtained at 4° C from 200 + 150 nl microseeded sitting drops with a 60 *μ*l reservoir consisting of 20% MPD, 0.1M Tris pH 8, and 7.5% PEG 5000 MME. Ternary crystals of p300‐HAT‐YF‐Δloop with CoA and CPI-090 (Fig. 2) were obtained at 4° C from 200 + 150 nl sitting drops with a 60 *μ*L reservoir consisting of 0.1 M CHES pH 9.5 and 30% w/v PEG 3000 [MCSG1 (MCSG) (purchased from Anatrace), condition A2]. As these were small crystals grown in MPD and PEG conditions, low-viscosity perfluoropolyether oil (purchased as “LVCO” from MiTeGen) was sufficient for cryoprotection (Riboldi‐Tunnicliffe and Hilgenfeld, 1999).

Following synchrotron data collection and reduction with XDS (Kabsch, 2010), the structures were solved by molecular replacement with Phaser (McCoy *et al.,* 2007), with 2 chains in the ASU, iterative cycles of rebuilding in Coot (Emsley and Cowtan, 2004) and refining in Refmac5 (Murshudov *et al.*, 1997). Coordinates and structure factors were deposited in the PDB with codes 6PGU (CPI-076) and 6PF1 (CPI-090). Data collection and refinement parameters are presented in Table II.

**TABLE II.** Crystallographic data collection and refinement statistics.

	CPI-076	CPI-090
Deposition code	6PGU	6PF1
Resolution range (Å)	71.78, 1.72	70.69, 2.32
Resolution range last shell (Å)	1.76, 1.72	2.44, 2.32
Space group	P212121	P212121
Unit cell (Å)	66.253, 97.599, 105.932	65.062, 90.634, 112.950
Unique reflections	69876	29312
Overall and last-shell completeness (%)	99.75	93.55, 90.32
Overall and last shell R_meas_	0.067	0.126, 0.726
Overall and last shell σ(I)	28.7 2.0	10.8, 2.3
Solution method	Molecular replacement	Molecular replacement
Refinement software	Refmac5	Refmac5
Rwork, Rfree	0.16727, 0.20627	0.18355, 0.24728
Number of atoms	5754	5315
Average of B factors	23.28	34.247
RMSD bond lengths	0.019	0.013
RMSD angles	2.033	1.645
Molprobity score and percentile rank	1.214, 98	1.154, 100

### A. Structure description—overall and binding mode

**FIG. 3.**
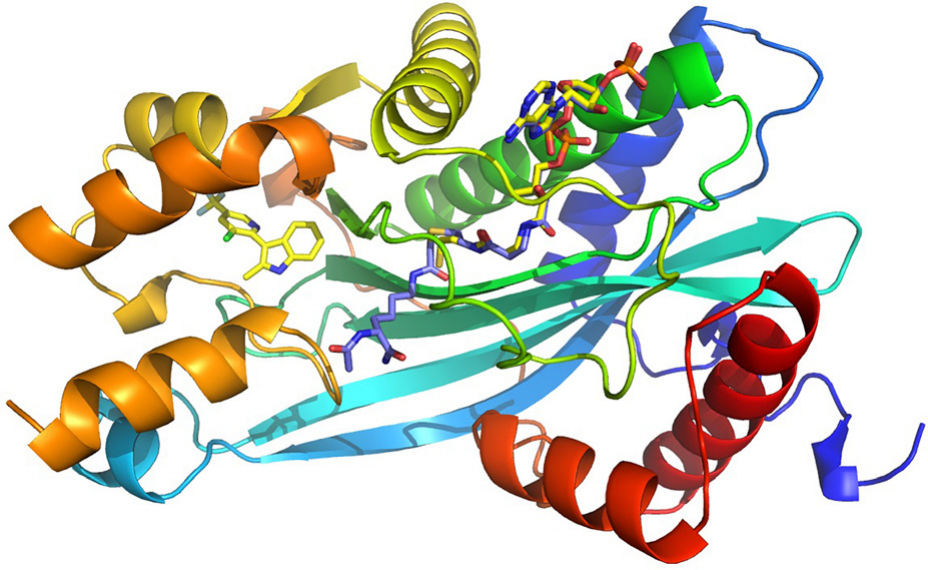
Lys-CoA dual substrate analog P300-HAT structure 3BIY superposed with the CPI-090 P300-HAT structure (yellow C atoms for CPI-090 and CoA). Lys's closest approach to CPI-090 is ∼8 Å.

**FIG. 4.**
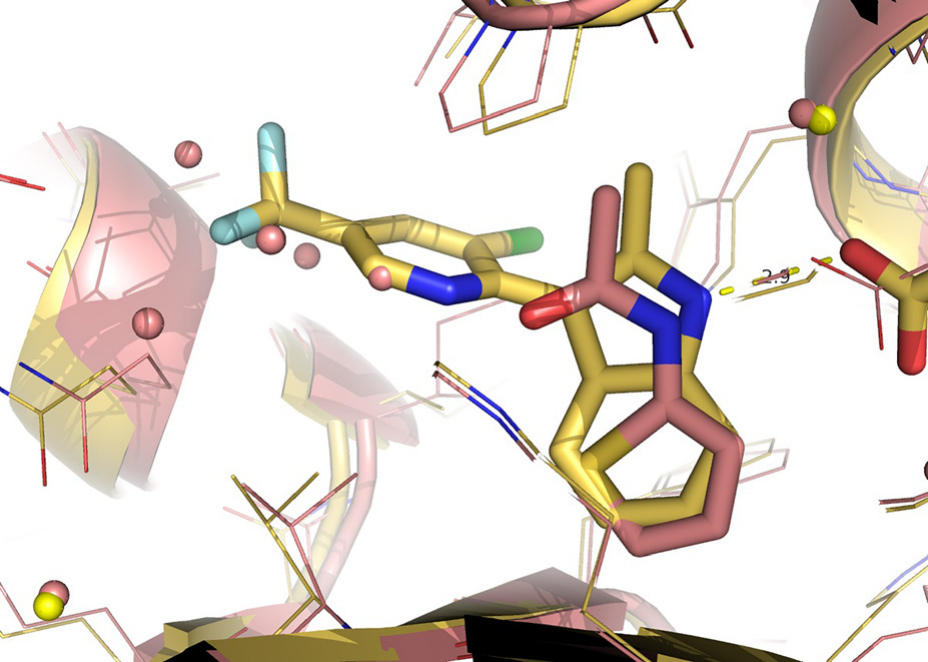
Comparing the binding modes of CPI-090 (yellow C-atoms) with CPI-076 (salmon C-atoms).

The overall structure is as expected from previous p300-HAT structures: a central β-sheet is composed of seven β-strands and surrounded by nine α-helices (Fig. 3). Despite their chemical differences, these new allosteric compounds bind in the same location (Fig. 4), ∼8 Å distant from the active site, between the α5 and α6 helices. While these compounds' MOA is formally modulated by the substrate and AcCoA, the structures show that the binding mode is distal from the substrate and AcCoA sites, which starts to rationalize the difference.

It is worth noting that in both cases, CoA was carried through purification and appeared fortuitously in the AcCoA binding site. So though these compounds are AcCoA modulated, there is obviously no direct competition for the AcCoA binding site.

**FIG. 5.**
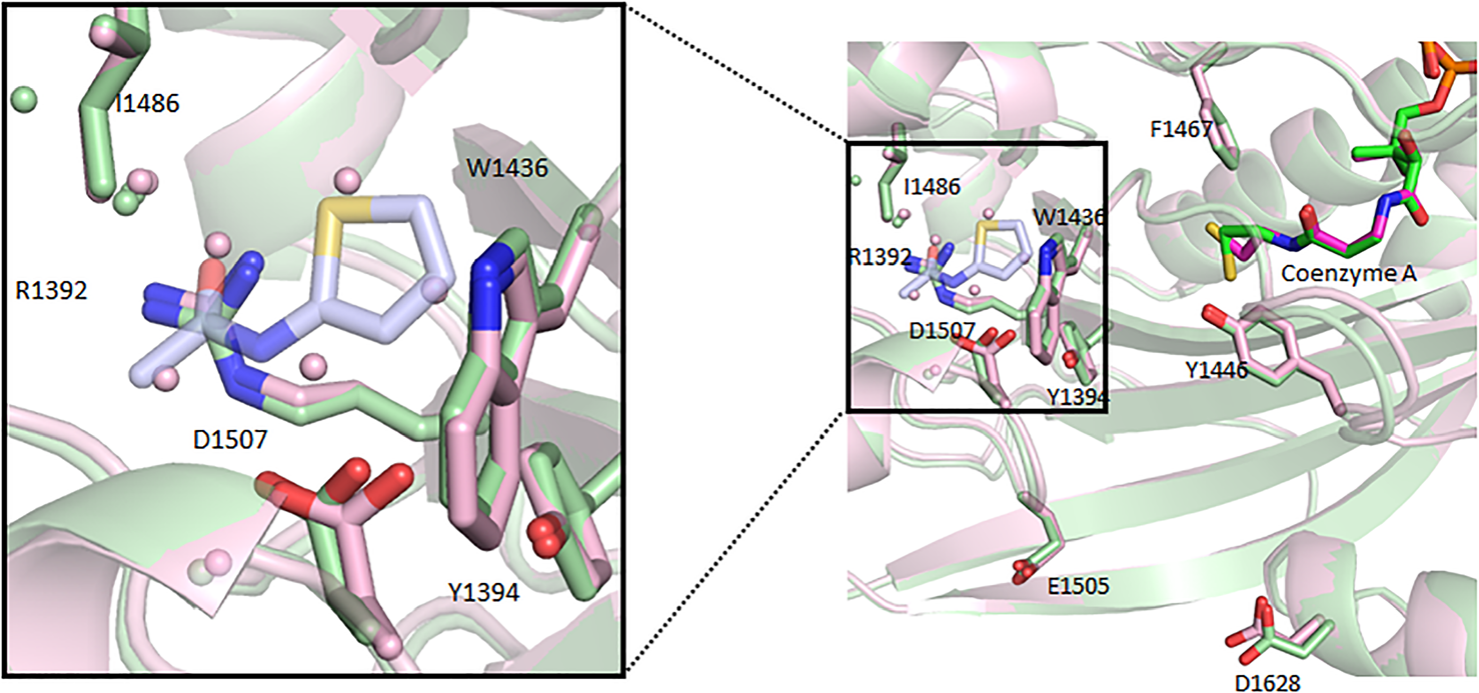
CPI-076-p300-HAT-CoA (light blue C atoms for the compound, green protein, and CoA) aligned with p300-HAT-CoA (pink protein and CoA) shows minimal perturbations in the protein backbone, as compound binding acts to displace five water molecules found in the CoA-only structure. Water molecules colored to match protein color.

There are few structural differences between these ternary compound/CoA/HAT structures and CoA/HAT binary structures. Comparing the HAT/CoA/CPI-090 structure with HAT/CoA (4PZR), the RMSD value was found to be 0.27 Å over 270 CA atoms. Essentially, the allosteric compounds displace water molecules (Fig. 5). The grounds for mechanistic competition with the substrate and AcCoA are not readily apparent. CPI-076 is secured in p300-HAT-CoA by hydrophobic interactions and a hydrogen bond with Asp1507 and also creates a water network out to Arg1392/Ile1486.

### B. SBDD

**FIG. 6.**
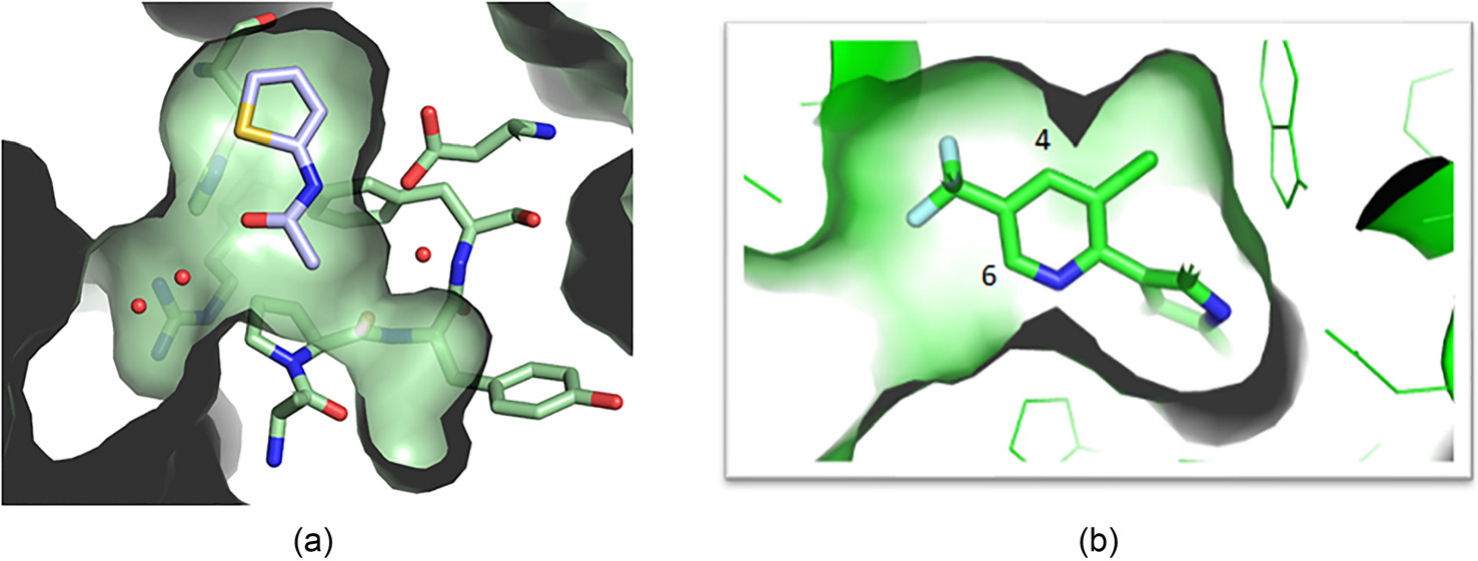
(a) CPI-076 offered two vectors for structure-based elaboration. (b) The indole compound CPI-090, a stronger HAT inhibitor, offered vectors for optimization from the 4, 5, and 6 positions of the pyridine ring.

With CPI-076's measured HAT-domain IC50 20 *μ*M, SBDD efforts focused on driving the biochemical activity. CPI-076's structure offered 2 potential vectors for compound elaboration [Fig. 6(a)]. However, derivatization failed to improve the compound activity from this starting point (data not shown). The indole compound CPI-090, a stronger HAT inhibitor at 0.31 *μ*M, offered vectors for optimization from the 4, 5, and 6 positions of its pyridine ring [Fig. 6(b)]. Derivatization yielded actionable SAR and improvement in the biochemical activity (data not shown), and so this series was selected for cell-based assays.

## IV. CLONING AND EXPRESSION OF FULL-LENGTH HUMAN P300 (FL-P300)

The sequence of 2414-residue full-length human p300 (FL-P300) was codon-optimized for insect cell expression and synthesized in three parts (Genscript). After assembly of the full-length sequence, the complete ORF was subcloned into a modified pTriEx vector (Novagen/EMD Millipore) to express FL-P300 with both an N-terminal, cleavable polyhistidine tag and C-terminal StrepII tag (HisTev-p300-Strep). Baculovirus was produced by co-transfection of the plasmid into Sf9 cells with linearized baculovirus BestBac2.0 (Expression Systems). The protein was expressed in Sf9 cells and purified with Talon Metal Affinity Resin (Clontech/Takara). The purified protein was concentrated to 1.6 mg/ml in storage buffer (50 mM Tris pH 8, 100 mM NaCl, 10% glycerol, and 0.5 mM TCEP).

## V. ASSAY COMPARISON

**FIG. 7.**
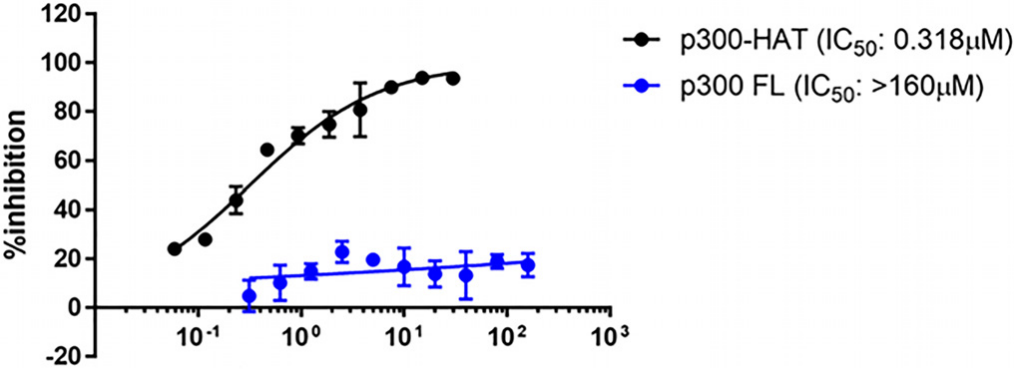
Inhibition of P300-HAT and P300-FL by CPI-090.

Moving the allosteric series compounds into cell-based assays assessing the ability of the compound to inhibit H3K18 acetylation in HCT116 cells delivered a setback to this allosteric series, as these compounds displayed such a large shift in cell-potency to be inactive. At this point in the program, quantities of highly purified FL P300 were more readily available (preparation described above), and so HAT inhibitors were retested against FL P300 under optimized conditions. A large shift relative to the P300-HAT catalytic domain assay was observed. CPI-090 does not inhibit the FL enzyme in our experiments (Fig. 7). The related compound CPI-076 had no measurable FL activity (Table III).

**TABLE III.** Comparison of allosteric inhibitor activities in various assays.

CPI-number	CPI-076	CPI-090
*Primary assays*		
P300 HAT SPA IC_50_	20 *μ*M	0.31 *μ*M
CBP HAT SPA IC_50_	39 *μ*M	0.74 *μ*M
H3K18Ac EC_50_		>30 *μ*M
FL p300 SPA IC_50_	Undetectable	>160 *μ*M

It became clear from the optimized FL assay that the (sub)micromolar inhibition observed against the catalytic HAT domain had been an artifact of using the HAT domain, which had been used because of initial limitations of quantity and purity in obtaining full-length P300.

## VI. CONCLUSIONS

The grounds for mechanistic modulation with the substrate and AcCoA in the HAT-only construct are not readily apparent. The structure of the p300-p53 complex (Ghosh *et al.,* 2019) (5xzc, at 10.7 Å) appeared at first to hint at a hypothesis, but further investigation ruled it out. The p300-p53 structure is noteworthy in that it contains a model of the p300 regulatory loop (residues ∼1520–1560), which has not to our knowledge been visualized before. While nothing in the p300-p53 complex structure appears to directly block access to the CPI-090/CPI-076 binding site, the regulatory loop packs against helix 7; in our HAT structures, helix 7 comes into contact with CPI-090/CPI-076 and is mobile (which lacks the loop). The activation loop was intact in our screening construct, though, which would seem to rule out the hypothesis of regulatory loop interference/mimicry.

The cellular and FL-P300 biochemical assays demonstrated that the allosteric HAT inhibitor series, which had been co-crystallized with a truncated form of the target enzyme and enhanced by SBDD, was artifactual. This series was ultimately abandoned, and series of compounds with alternative mechanisms of action were prioritized for our medicinal chemistry campaign. The results of this case study demonstrate that hits from partial enzymes may be artifactual, not translating into cellular efficacy, despite compelling support from DSF and crystallographic experiments. The lesson learned is to prioritize the use of full-length enzymes when possible in biochemical assays early in compound screening or follow-up and to carefully examine any anomalies or discrepancies between assay results. The challenges in obtaining FL-P300 led us to rely on a truncated form of the target enzyme and a focus on this series, which ultimately lacked pharmacological relevance.
